# Unveiling the dual role of CD3G: a diagnostic biomarker for depression and its oncogenic implications

**DOI:** 10.3389/ebm.2025.10599

**Published:** 2025-10-01

**Authors:** Hai Gao, Ting Wu, Jihui Xue, Jing Liu, Dongmei Wen, Guanwei Huang

**Affiliations:** ^1^ Nutrition Department, Xiamen Xianyue Hospital, Xiamen, Fujian, China; ^2^ Hospital Infection Control Department, Xiamen Xianyue Hospital, Xiamen, Fujian, China; ^3^ Xianyue Hospital Affiliated with Xiamen Medical College, Xiamen, Fujian, China; ^4^ Fujian Psychiatric Center, Xiamen, Fujian, China; ^5^ Fujian Clinical Research Center for Mental Disorders, Xiamen, Fujian, China; ^6^ Department of Infectious Diseases, The First Affiliated Hospital of Xiamen University, Xiamen, Fujian, China; ^7^ Public health Department, Xiamen Xianyue Hospital, Xiamen, Fujian, China; ^8^ Operation Management Department, Xiamen Maternnal and Child Health Hospital, Xiamen, Fujian, China

**Keywords:** depression, immune dysregulation, CD3G, diagnostic marker, inflammatory pathways

## Abstract

Depression has been increasingly associated with immune system dysregulation. This study investigates the potential of CD3 Gamma Subunit of T-Cell Receptor Complex (CD3G) as a diagnostic marker for depression, while also examining its role across various cancer types. Comparative analyses of immune cell infiltration and pathway activities were conducted using single-sample Gene Set Enrichment Analysis (ssGSEA) on datasets GSE98793. Depression patients were defined based on clinical diagnoses and compared to healthy controls (HC) without any psychiatric disorders. Differentially expressed genes (DEGs) were identified, followed by weighted gene co-expression network analysis (WGCNA), least absolute shrinkage and selection operator (LASSO) and logistic regression to pinpoint independent diagnostic markers. Functional enrichment analyses were performed to explore the biological implications of CD3G expression in depression. Pan-cancer analyses were also conducted to investigate CD3G’s role in cancer. Depression patients exhibited significant decreases in CD8 T cells, cytotoxic cells, T cells, T helper cells, Tgd, and Th2 cells, with increased levels of dendritic cells and neutrophils compared to HC. Immune pathway activities showed increased antimicrobial, chemokine, cytokine, and TNF family member activities, with decreased TCR signaling activity in depression patients. CD3G was identified as a key immune-related gene and independent diagnostic marker for depression, validated by GSE76826 dataset. Low CD3G expression in depression was associated with enhanced immune response and inflammatory pathways. In pan-cancer analysis, CD3G was upregulated in numerous cancers and correlated with immune cell infiltration and oncogenic pathways. The study highlights significant dysregulation in immune cell infiltration and pathway activities in depression, with CD3G emerging as a critical immune-related gene and potential diagnostic marker. CD3G’s role in immune modulation and cancer underscores its relevance in both depression and oncology, suggesting potential therapeutic targets and prognostic indicators.

## Impact statement

The findings of this study are expected to enhance our understanding of the immune mechanisms underlying depression and identify CD3G as a critical biomarker for diagnosis. Moreover, the dual role of CD3G in both depression and cancer underscores its potential as a therapeutic target, offering new insights into the intersection of neuroimmune and oncogenic pathways. This research not only provides a novel perspective on the pathophysiology of depression but also paves the way for innovative diagnostic and therapeutic approaches.

## Introduction

Depression is a debilitating mental health disorder that affects millions of people worldwide, contributing significantly to global morbidity and disability [1, 2]. Traditionally, depression has been primarily understood through a neurochemical lens, focusing on imbalances in neurotransmitters such as serotonin, dopamine, and norepinephrine [3, 4]. However, emerging evidence suggests that depression is also closely linked to immune system dysregulation, indicating a more complex pathophysiology involving neuroimmune interactions [5].

Recent studies have highlighted the role of immune cells and inflammatory pathways in the development and progression of depression [6, 7]. Immune cell infiltration and the activation of specific immune pathways have been observed in patients with depression, suggesting that immune dysregulation may contribute to the onset and maintenance of depressive symptoms [8, 9]. This paradigm shift opens new avenues for identifying novel biomarkers and therapeutic targets, potentially leading to more effective diagnosis and treatment strategies. One such promising biomarker is CD3G, a gene encoding the gamma chain of the CD3 complex, which is crucial for T cell receptor signaling and T cell function [10–12]. Previous research has implicated CD3G in various immune-related processes, and its dysregulation has been observed in several diseases, including autoimmune disorders and cancers [13–15]. However, its specific role in depression remains largely unexplored.

This study aims to investigate the potential of CD3G as a diagnostic marker for depression by analyzing immune cell infiltration and pathway activities in depression patients compared to healthy controls (HC). We conducted comprehensive bioinformatics analyses using ssGSEA on publicly available datasets (GSE98793) to identify key immune-related pathways. Additionally, Weighted Gene Co-expression Network Analysis (WGCNA), Least Absolute Shrinkage and Selection Operator (LASSO) regression, and logistic regression were employed to pinpoint independent diagnostic markers. Functional enrichment analyses were performed to elucidate the biological implications of CD3G expression in depression. Furthermore, we extended our investigation to a pan-cancer analysis to examine CD3G’s role across various cancer types, given its known involvement in immune modulation.

The findings of this study are expected to enhance our understanding of the immune mechanisms underlying depression and identify CD3G as a critical biomarker for diagnosis. Moreover, the dual role of CD3G in both depression and cancer underscores its potential as a therapeutic target, offering new insights into the intersection of neuroimmune and oncogenic pathways. This research not only provides a novel perspective on the pathophysiology of depression but also paves the way for innovative diagnostic and therapeutic approaches.

## Materials and methods

### Depression-related dataset acquisition

The depression-associated dataset was acquired from the NCBI GEO database (https://www.ncbi.nlm.nih.gov/geo/) [16]. Using the search term “depression” on the GEO homepage, the dataset was filtered based on specific criteria: 1) inclusion of blood samples from both healthy individuals and individuals with depression; 2) availability of raw data in the dataset; and 3) a minimum sample size of 10. Two datasets, GSE98793 and GSE76826, were ultimately chosen (Table 1). GSE98793 includes 64 healthy controls and 128 individuals with depression, while GSE76826 includes 12 healthy controls and 20 individuals with depression. A sample size calculation was not explicitly performed; however, the selected datasets met the predefined criteria to ensure sufficient statistical power for downstream analysis. Microarray data and platform annotation files were retrieved using the GEOquery package (v.2.76.0) in R software. Gene ID conversion was conducted on the downloaded matrix files according to the annotation files, removing probes with missing or duplicate annotations. Data analysis was performed using the limma package (v.3.64.0) in R software, with microarray data normalization carried out using the normalizeBetweenArrays function within the limma package (v.3.64.0).

**TABLE 1:** Information on depression-related datasets used in the current study.

GEO ID	Platform	Healthy controls	Depression	Source	Application
GSE98793	GPL570	64	128	Blood	Analysis
GSE76826	GPL17077	12	20	Blood	Validation

### Evaluation of immune microenvironment

To evaluate the differential abundance of immune cell subsets and immune response pathways between the healthy control (HC) and depression cohorts, we implemented single-sample Gene Set Enrichment Analysis (ssGSEA). The gene sets employed for the quantification of various immune cell populations were derived from established literature [17]. The xCell algorithm was utilized to assess the immune score levels between the HC and depression groups. Concurrently, we retrieved genomic datasets pertinent to immune response pathways from the ImmPort repository (http://www.immport.org) [18]. Immune pathways-related genes (IPGs) were also obtained from the ImmPort database. Pearson correlation analysis was conducted to determine the relationship between CD3G expression and the composition of immune cell subsets, as well as immune response pathways.

### Utilizing WGCNA to identify immune-related significant modules

Co-expression networks were established using the WGCNA package (v.1.6.0) in R software [19]. The determination of an appropriate soft threshold β with a correlation coefficient R^2^ > 0.9 was conducted based on GSE98793 expression profiles. Subsequently, the adjacency matrix of the GSE98793 microarray expression profile was constructed following weighted calculation, and then converted to a topological overlap matrix using the TOM overlap calculation formula. The correlation between each gene module and the sample phenotype was calculated, leading to the identification of the gene module exhibiting the strongest correlation with the immune score as the pivotal module.

### Identification of signature gene for depression

We employed the limma package (v.3.64.0) to identify differentially expressed genes (DEGs) between the HC and depression cohorts, defining DEGs as genes with an adjusted p-value <0.05. Utilizing the Venn tool, we generated a Venn diagram to depict the overlap of genes from DEGs, IPGs, and significant modules identified via WGCNA. These overlapping genes were denoted as pivotal immune-related genes. LASSO regression was then conducted to unveil signature genes from the pivotal immune-related gene set, which were further refined through Cox regression analysis.

### Analysis of the potential biological functions of the CD3G gene

Depression samples in the GSE98793 dataset were stratified into CD3G-low and CD3G-high cohorts based on the median CD3G expression levels. Differentially expressed genes (DEGs) between the CD3G-low and CD3G-high cohorts were determined using the limma package (v.3.64.0), with an adjusted p-value threshold of <0.05. Visualization of DEGs was accomplished through the ComplexHeatmap package (v.2.24.0). Gene Ontology (GO) and Kyoto Encyclopedia of Genes and Genomes (KEGG) enrichment analyses were conducted using the clusterProfiler R package (v.4.16.0), with the results illustrated via the ggplot2 package (v.3.4.4). Additionally, the ggplot2 package facilitated the visualization of variations in immune infiltrate levels and immune pathways between the CD3G-low and CD3G-high cohorts.

### Pan-cancer analysis of CD3G

The gene expression data for CD3G across 33 types of cancer were obtained from The Cancer Genome Atlas (TCGA) database (https://portal.gdc.cancer.gov/) [20]. This dataset includes normalized RNA-Seq data for various tumor and adjacent normal tissues. Clinical data, including overall survival information, were also retrieved to assess prognostic significance. Box plots were generated to compare the expression levels of CD3G between tumor and normal tissues for each cancer type. Statistical significance was determined using the Wilcoxon rank-sum test, with a p-value threshold of <0.05 considered significant. The prognostic value of CD3G expression in different cancers was evaluated using univariate Cox regression analysis. Forest plots were used to visualize the hazard ratios (HR) and 95% confidence intervals (CI) for overall survival across the 33 cancer types. The relationship between CD3G expression and immune cell infiltration was analyzed using the single-sample Gene Set Enrichment Analysis (ssGSEA) algorithm (v.1.44.5). The ssGSEA scores for various immune cell types were computed for each tumor sample. Correlation coefficients between CD3G expression and immune cell infiltration scores were calculated using Spearman correlation test. A heatmap was constructed to display the correlation coefficients. Box plots and forest plots were generated using the ggplot2 package (v.3.4.4), while heatmaps were created using the ComplexHeatmap package (v.2.24.0).

### Gene set variation analysis (GSVA)

The z-score algorithm in the R-package GSVA (v.1.44.5) was utilized to integrate CD3G gene expression as a reflection of pathway activity across 14 functional state gene sets. Z-scores were computed individually for each gene set, followed by calculating the Pearson correlation analysis to assess the statistical correlation between genes and each gene set z-score [21, 22].

### Drug sensitivity analysis

The association between CD3G gene expression in the GDSC1 and GDSC2 databases and the IC50 of chemotherapeutic drugs was assessed through Spearman correlation analysis [23]. A negative correlation indicates that higher gene expression corresponds to increased drug sensitivity in the cell line, while a positive correlation suggests that elevated gene expression leads to greater drug resistance.

### Statistical analysis

All statistical analyses were conducted in the R programming environment (version 4.2.1). Wilcoxon rank-sum tests compared CD3G expression between disease and normal groups. Univariate Cox regression evaluated prognostic significance. Spearman correlation analysis linked CD3G expression to immune infiltration.

## Results

### Secondary outcome: immune cell infiltration patterns and pathway activity in depression

#### Comparative analysis of immune cell infiltration and pathway activity

**FIGURE 1:**
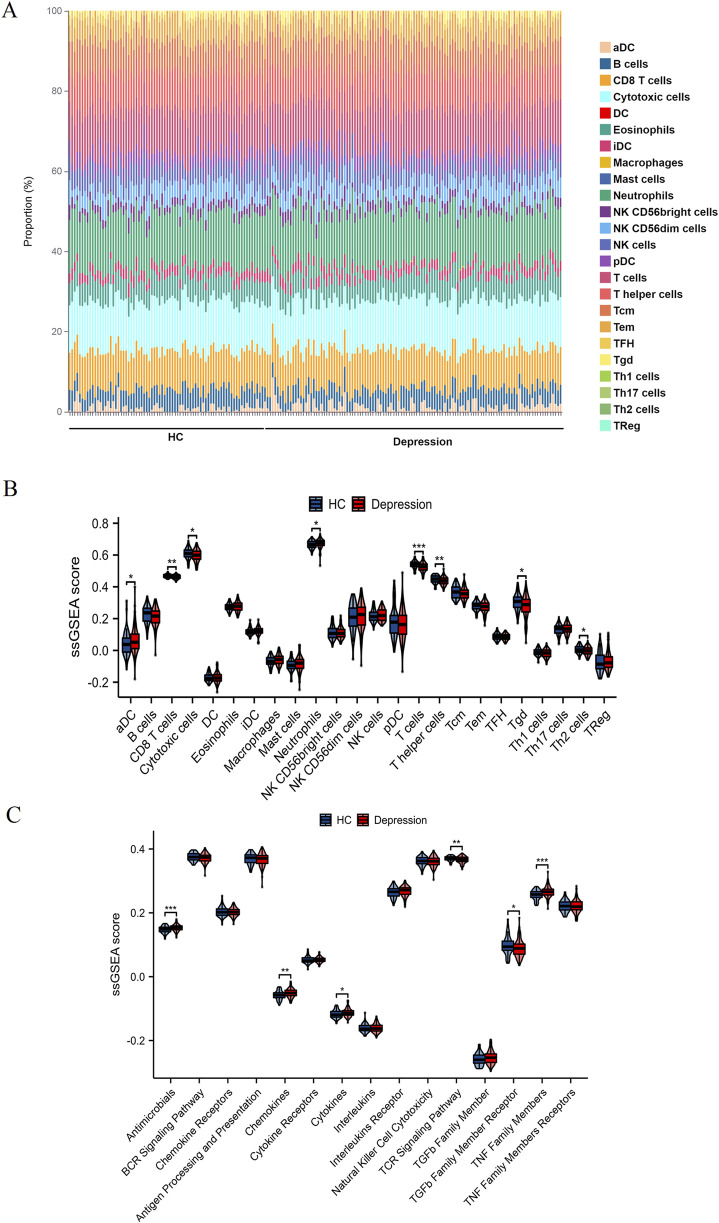
Comparative analysis of immune cell infiltration and immune pathway activity. **(A)** Proportional distribution of various immune cell types in HC and depression patients. Each bar represents a single sample, with different colors indicating different immune cell subsets. **(B)** ssGSEA scores illustrating the differences in the infiltration of specific immune cell types between HC (blue) and depression patients (red). **(C)** ssGSEA scores showing the activity levels of various immune-related pathways in HC (blue) and depression patients (red). Statistically significant differences are marked with asterisks (*P < 0.05, **P < 0.01, ***P < 0.001).

Figures 1A,B reveals distinct immune cell distribution patterns between HC and depression patients. Notably, the depression group exhibited marked reductions in CD8 T cells, cytotoxic cells, T cells, T helper cells, Tgd, and Th2 cells compared to HC (all P < 0.05), while dendritic cells (aDC) and neutrophils were significantly elevated (P < 0.05). Consistent with these cellular changes, ssGSEA analysis (Figure 1C) demonstrated divergent pathway activity profiles: antimicrobials, chemokine, cytokine, and TNF family members showed pronounced upregulation in depression (P < 0.05), whereas TCR signaling and TGFb family member receptor activities were substantially suppressed (P < 0.05). Collectively, these findings underscore systemic immune dysregulation in depression, characterized by altered cellular infiltration and pathway activation.

### Primary outcome: immune score-associated gene modules and CD3G as a diagnostic biomarker

#### WGCNA reveals immune score-associated gene modules

**FIGURE 2:**
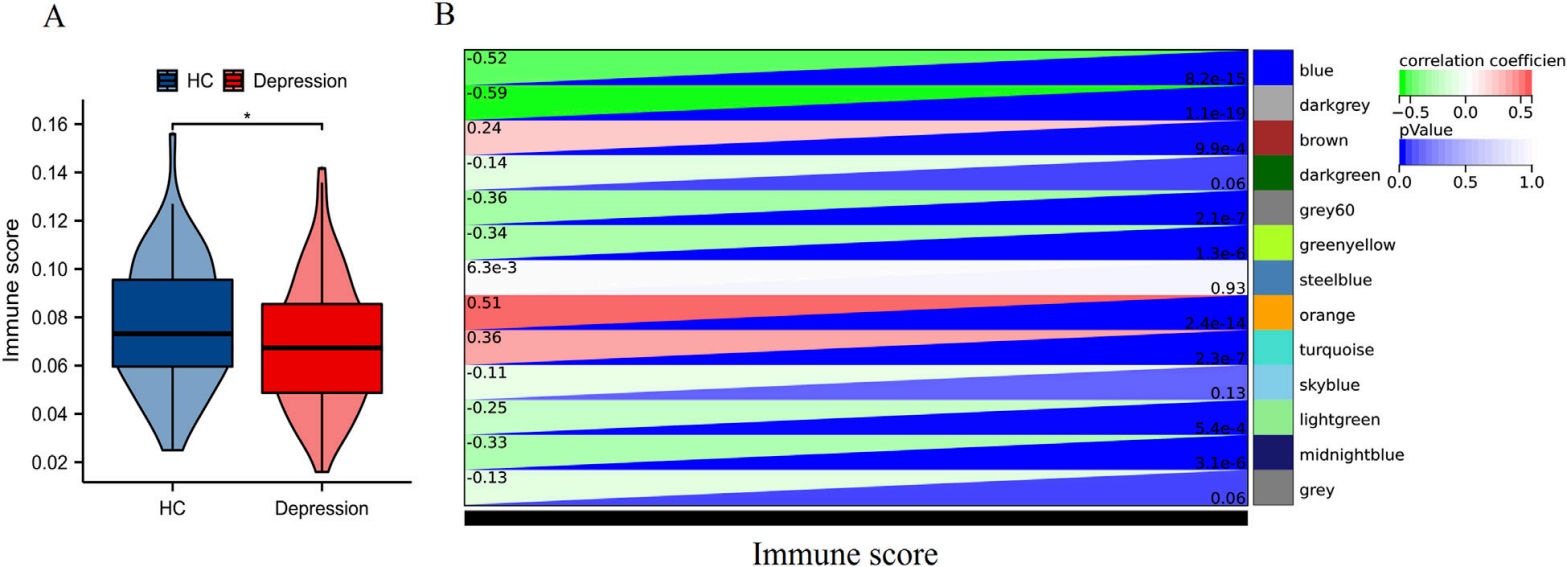
WGCNA based on immune scores. **(A)** Distribution of immune scores between HC and depression patients. *P < 0.05. **(B)** Heatmap of module-trait relationships displaying the correlation between module eigengenes and immune scores.

As illustrated in Figure 2A, immune scores were significantly diminished in depression patients versus HC (P < 0.05). WGCNA identified three critical modules (Figure 2B) correlated with immune scores: the blue and darkgrey modules showed strong negative correlations (r = −0.52 and −0.59, respectively), whereas the orange module displayed robust positive correlation (r = 0.51). These results implicate immune-related gene network dysregulation as a potential contributor to depression pathogenesis.

#### CD3G as a diagnostic biomarker for depression

**FIGURE 3:**
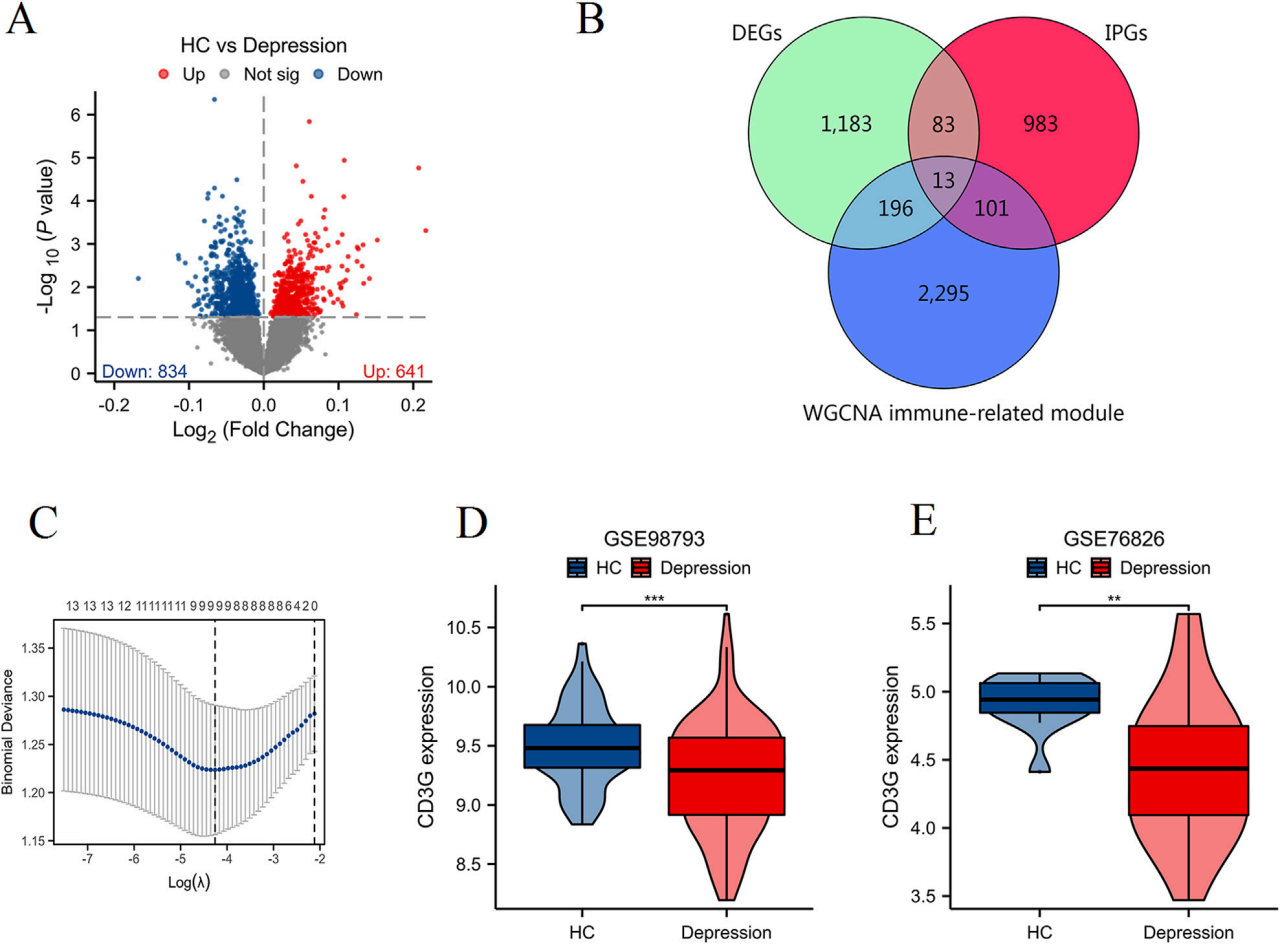
Identification and validation of key immune-related gene CD3G in depression. **(A)** Volcano plot showing DEGs between HC and depression patients. **(B)** Venn diagram illustrating the intersection of DEGs, IPGs, and genes from significant WGCNA modules. **(C)** LASSO regression analysis for feature selection. Expression levels of CD3G in the GSE98793 dataset **(D)** and GSE76826 dataset **(E)**. ***P < 0.001, **P < 0.01.

Volcano plot analysis (Figure 3A) identified 1,475 DEGs (834 downregulated, 641 upregulated) in depression. Intersection of DEGs, immune pathway genes (IPGs), and WGCNA module genes (Figure 3B) yielded 13 immune-related hub genes. LASSO regression (Figure 3C) and subsequent logistic regression uniquely identified CD3G as an independent diagnostic marker (P < 0.038) (Table 2). Consistent downregulation of CD3G was validated in both discovery (GSE98793, Figure 3D, P < 0.001) and replication cohorts (GSE76826, Figure 3E, P < 0.01), solidifying its role as a robust diagnostic indicator.

**TABLE 2:** The results of Logistic analysis.

Characteristics	Total(N)	Univariate analysis		Multivariate analysis	
		Odds ratio (95% CI)	P value	Odds ratio (95% CI)	P value
VEGFC	192	4.447 (1.698–11.649)	**0.002**	2.710 (0.906–8.109)	0.075
CD3G	192	0.262 (0.121–0.566)	**<0.001**	0.385 (0.156–0.949)	**0.038**
PRKCQ	192	0.467 (0.199–1.098)	0.081	2.939 (0.820–10.537)	0.098
CXCR6	192	0.440 (0.232–0.834)	**0.012**	0.508 (0.238–1.088)	0.081
TNFSF13	192	2.920 (1.381–6.177)	**0.005**	2.304 (0.890–5.963)	0.085
S100B	192	0.519 (0.312–0.864)	**0.012**	0.709 (0.399–1.260)	0.241
S100P	192	1.316 (1.023–1.694)	**0.032**	1.215 (0.910–1.623)	0.187
TRAV12-2	192	0.265 (0.107–0.654)	**0.004**	0.521 (0.185–1.467)	0.217
TRGJ1	192	0.378 (0.165–0.866)	**0.022**	0.537 (0.203–1.423)	0.211

Bold type indicates a p-value less than 0.05.

### Exploratory outcomes: CD3G expression subgroups and pan-cancer implications

#### CD3G expression subgroups reveal immune pathway divergence

**FIGURE 4:**
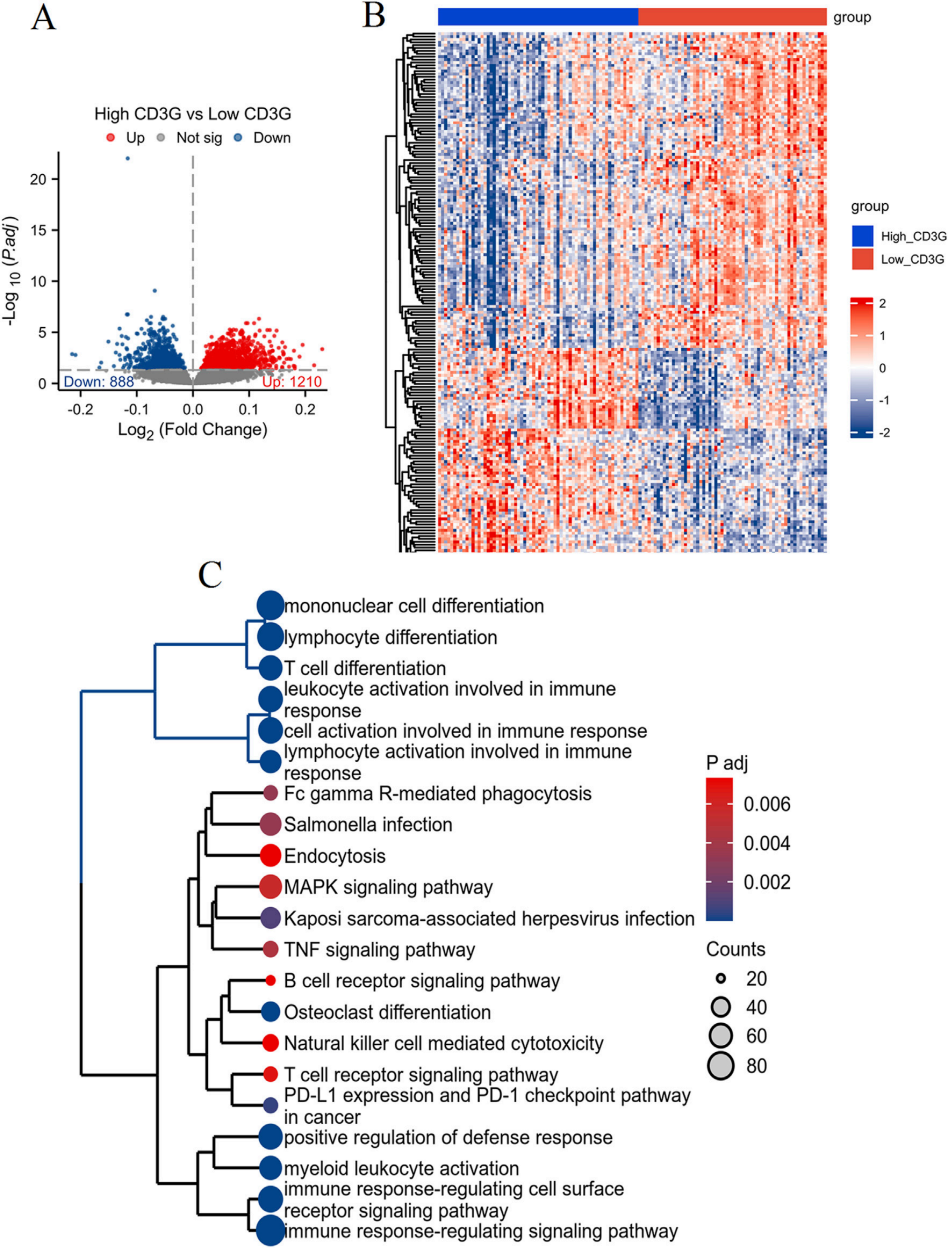
Differential expression and enrichment analysis based on CD3G expression levels in depression. **(A)** Volcano plot showing DEGs between high CD3G and low CD3G depression subgroups. **(B)** Heatmap of hierarchical clustering analysis of DEGs between high CD3G and low CD3G expression groups. **(C)** Functional enrichment tree plot displaying the significantly enriched pathways and processes associated with DEGs between high and low CD3G depression subgroups.

**FIGURE 5:**
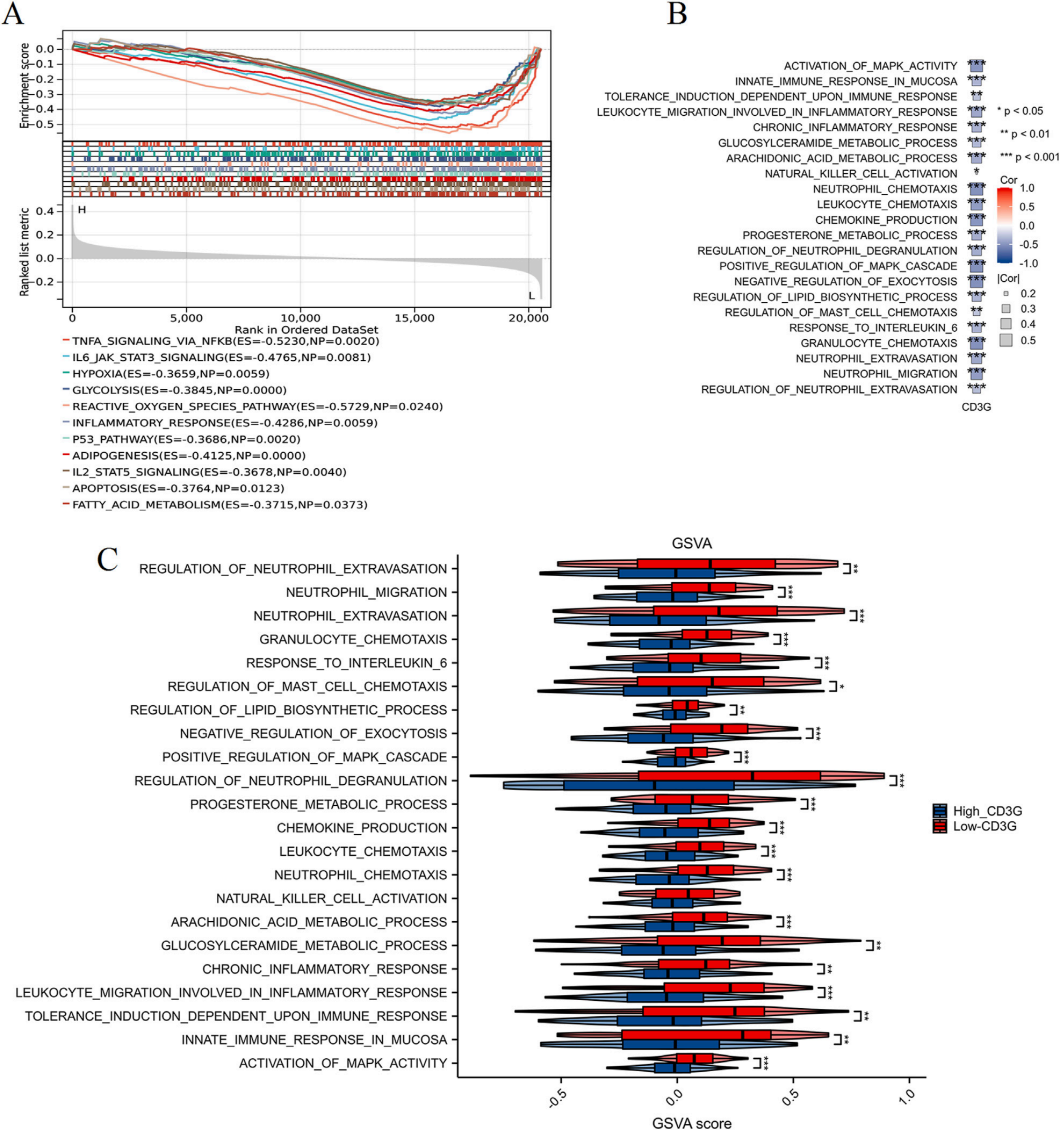
Enrichment analysis of high and low CD3G expression subgroups in depression. **(A)** GSEA plot comparing high CD3G and low CD3G expression subgroups. **(B)** Heatmap showing the correlation between CD3G expression and pathway activity based on GSVA. **(C)** GSVA scores comparing pathway activity between high and low CD3G expression subgroups. ***P < 0.001, **P < 0.01, *P < 0.05.

Comparative transcriptomic profiling (Figures 4A,B) between high- and low-CD3G depression subgroups identified 2,098 DEGs (888 downregulated, 1,210 upregulated). Functional enrichment analysis (Figure 4C) highlighted the enriched immune pathways: mononuclear cell differentiation, lymphocyte differentiation, T cell differentiation, leukocyte activation, and several immune response-related pathways such as the T cell receptor signaling pathways, natural killer cell mediated cytotoxicity, and PD-L1 expression and PD-1 checkpoint pathway in cancer. GSEA/GSVA (Figures 5A–C) further confirmed elevated inflammatory signatures (NFKB/STAT3 signaling, hypoxia, oxidative stress) in the low-CD3G subgroup (P < 0.05), with CD3G expression inversely correlating with chemokine production and leukocyte migration pathways.

#### Immune microenvironment differences by CD3G status

**FIGURE 6:**
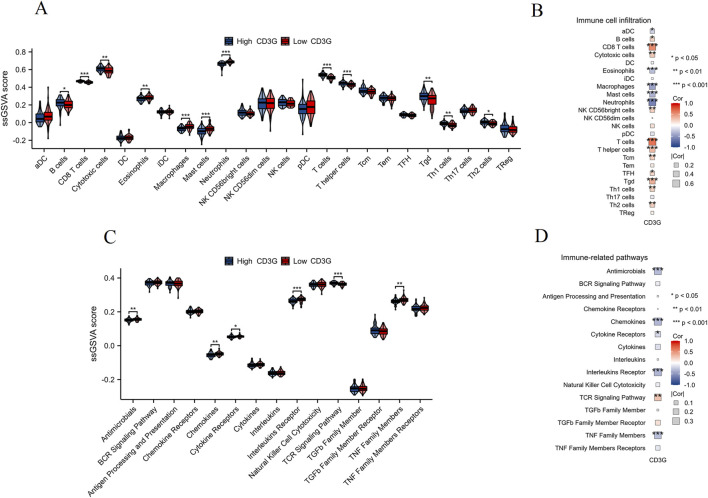
Immune cell infiltration and immune pathway activity analysis in CD3G-related subgroups. **(A)** ssGSEA scores comparing the infiltration levels of various immune cell types between high CD3G (blue) and low CD3G (red) expression subgroups. **(B)** Heatmap showing the correlation between CD3G expression and immune cell infiltration. **(C)** ssGSEA scores comparing the activity of immune-related pathways between high CD3G (blue) and low CD3G (red) expression subgroups. **(D)** Heatmap depicting the correlation between CD3G expression and the activity of immune-related pathways. ***P < 0.001, **P < 0.01, *P < 0.05.

ssGSEA profiling (Figures 6A,C) revealed contrasting immune cell infiltration patterns: the high-CD3G subgroup had abundant adaptive immune cells (B/T cells, Th1/Th2 subsets), whereas the low-CD3G group showed dominance of innate immune populations (neutrophils, macrophages, mast cells) (P < 0.05). Correlation heatmaps (Figures 6B,D) strengthened these associations, with CD3G expression positively linked to TCR signaling but negatively associated with cytokine/TNF pathways, suggesting CD3G as a master regulator of immune polarization in depression.

#### Pan-cancer implications of CD3G dysregulation

**FIGURE 7:**
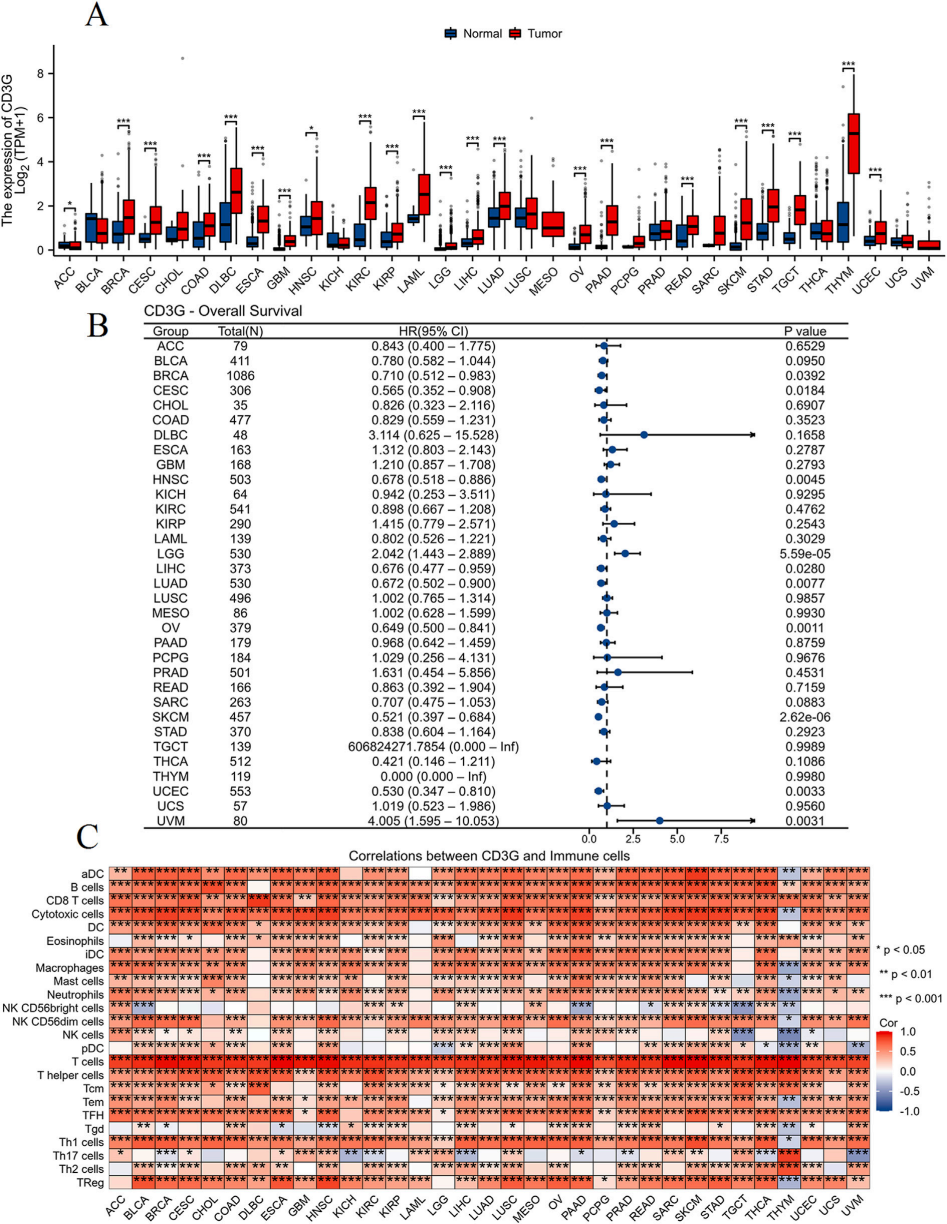
Pan-cancer analysis of CD3G. **(A)** Box plot comparing CD3G expression levels between normal (blue) and tumor (red) tissues across various types of cancer. **(B)** Forest plot displaying the hazard ratios (HR) with 95% confidence intervals (CI) and P-values for overall survival (OS) associated with CD3G expression in different cancer types. **(C)** Heatmap showing the correlation between CD3G expression and infiltration levels of various immune cells across multiple cancer types.

Cross-cancer analysis (Figure 7A) demonstrated widespread CD3G upregulation in 20 malignancies (e.g., BRCA, LUAD; P < 0.001) but downregulation in ACC (P < 0.05). Figure 7B shows the association of CD3G expression with overall survival (OS) across different types of cancer. CD3G expression was associated with the OS in BRCA (P = 0.0392), CESC (P = 0.0184), HNSC (P = 0.0045), LGG (P < 0.001), LIHC (P = 0.028), LUAD (P = 0.0077), OV (P = 0.0011), SKCM (P < 0.001), UCEC (P = 0.0033), and UVM (P = 0.0031). CD3G expression positively correlated with infiltration levels of most of immune cells, such as B cells, CD8 T cells, cytotoxic cells, neutrophils, Th1 cells (P < 0.05), across multiple cancers, positioning it as a pan-cancer immune modulator (Figure 7C).

#### CD3G links oncogenesis and drug sensitivity

**FIGURE 8:**
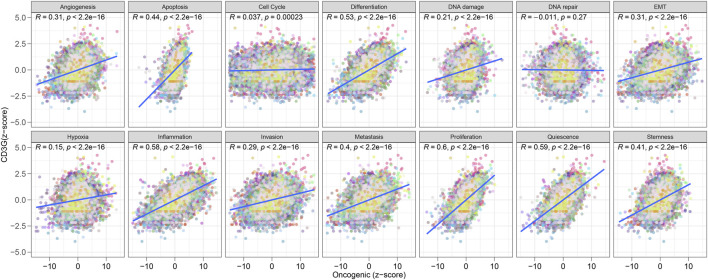
Correlation between CD3G expression and oncogenic pathways.

**FIGURE 9:**
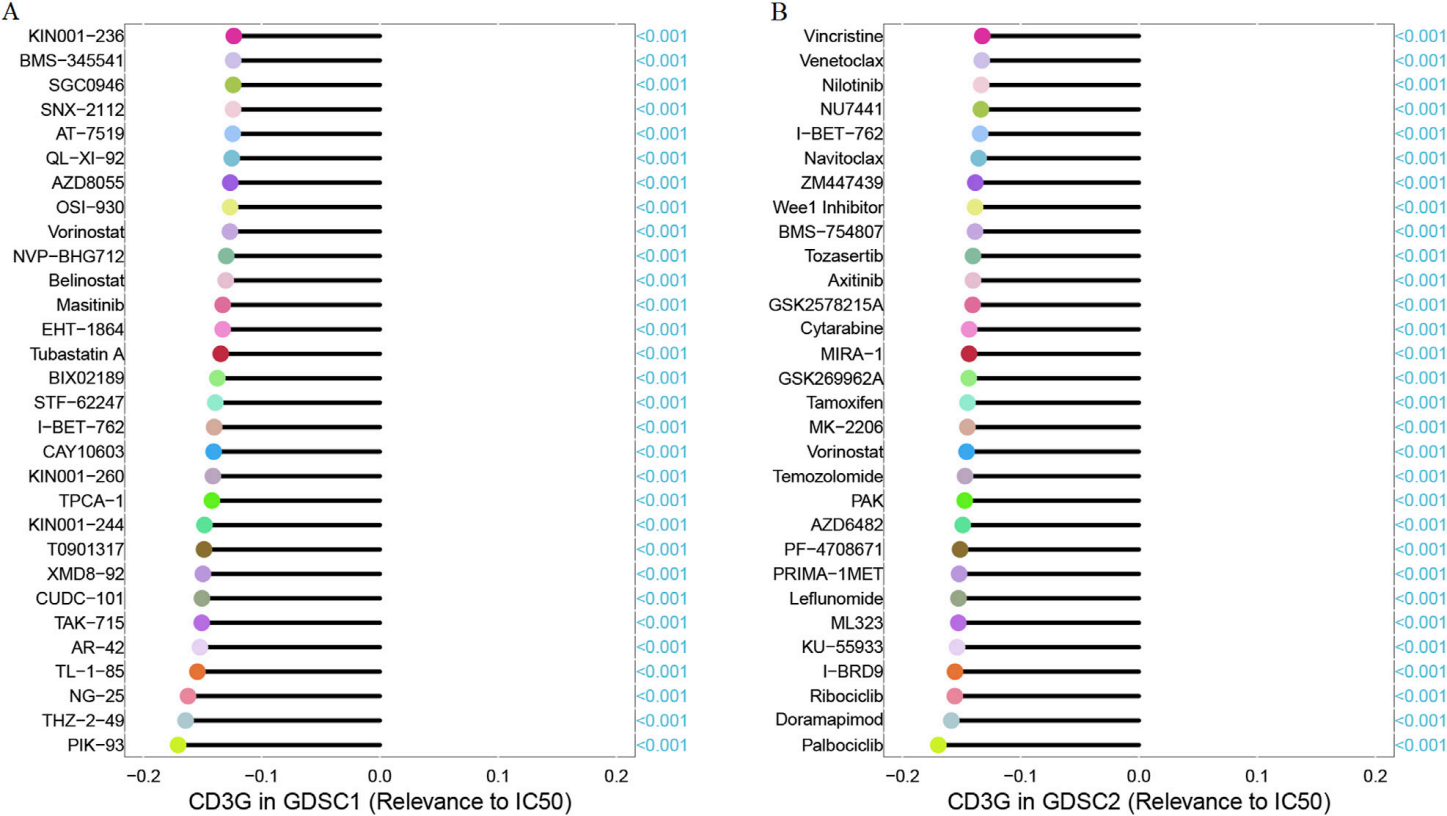
Correlation between CD3G expression and drug sensitivity (IC50) in GDSC1 **(A)** and GDSC2 **(B)** databases.

As shown in Figure 8, significant positive correlations were observed between CD3G expression and several oncogenic processes, including: Angiogenesis (R = 0.31, P < 2.2e-16), Apoptosis (R = 0.44, P < 2.2e-16), Differentiation (R = 0.53, P < 2.2e-16), DNA Damage (R = 0.21, P < 2.2e-16), EMT (Epithelial-Mesenchymal Transition) (R = 0.31, P < 2.2e-16), Hypoxia (R = 0.15, P < 2.2e-16), Inflammation (R = 0.58, P < 2.2e-16), Invasion (R = 0.29, P < 2.2e-16), Metastasis (R = 0.4, P < 2.2e-16), Proliferation (R = 0.6, P < 2.2e-16), Quiescence (R = 0.59, P < 2.2e-16), and Stemness (R = 0.41, P < 2.2e-16). These results demonstrate that high CD3G expression is strongly associated with the activation of many oncogenic pathways, reflecting its potential role in promoting cancer progression. In addition, Figure 9A shows the Spearman correlation analysis of CD3G gene expression with the half-maximal inhibitory concentration (IC50) of various antagonists in the GDSC1 database. Notably, CD3G expression was negatively correlated with the IC50 values for multiple drugs, including PIK-93, THZ-2-49, NG-25, TL-1-85, AR-42, etc (all P < 0.001), indicating that higher CD3G expression is associated with increased sensitivity to these drugs. Figure 9B depicts the Spearman correlation analysis of CD3G gene expression with the IC50 values in the GDSC2 database. The analysis includes 30 different drugs, with significant negative correlations for CD3G expression observed for drugs such as Vincristine, Venetoclax, Nilotinib, NU7441, I-BET-762, etc (all P < 0.001). These results suggest that elevated CD3G expression levels increase the sensitivity of cancer cell lines to these anticancer agents.

## Discussion

In this study, we explored the dual role of CD3G as a potential diagnostic biomarker for depression and its oncogenic implications across various cancer types. Our findings reveal significant dysregulation of immune cell infiltration and pathway activities in patients with depression, with CD3G emerging as a critical immune-related gene.

In recent years, a growing body of research has underscored the significance of immune processes in the onset of depressive disorder [24]. Our results confirm and extend previous studies that have linked depression with immune system dysregulation. For instance, the observed decrease in CD8 T cells, cytotoxic cells, and various T cell subsets (T helper cells, Tgd, Th2) alongside an increase in dendritic cells and neutrophils is consistent with earlier research indicating altered immune profiles in depression [25, 26]. The increased activities of antimicrobial, chemokine, cytokine, and TNF family member pathways, coupled with decreased TCR signaling pathway, further underscore the complex interplay between immune response and depressive disorders. These findings align with the concept of inflammation and immune activation playing a crucial role in the pathophysiology of depression [27, 28].

Building upon the identification of CD3G as an independent diagnostic marker for depression, it is essential to delve deeper into the mechanistic underpinnings of its association with immune infiltration and inflammatory pathways. Our analyses revealed that lower expression levels of CD3G correlate with an upregulation of immune response and inflammatory pathways. This observation aligns with the growing body of evidence suggesting that immune dysregulation plays a pivotal role in the pathophysiology of depression. The role of CD3G in TCR signaling is well-documented, with CD3G being a critical component of the CD3 complex essential for T-cell activation and immune response modulation [29]. Its reduced expression, as observed in our study, may lead to impaired T-cell function, thereby triggering compensatory mechanisms that enhance systemic inflammation. This is supported by studies indicating that T-cell dysfunction can result in a skewed cytokine production profile, favoring pro-inflammatory states [26]. Furthermore, the association between low CD3G expression and increased immune response in depression might be indicative of an underlying chronic inflammatory state. Chronic inflammation has been implicated in the pathogenesis of depression, with elevated levels of inflammatory markers such as interleukin-6 and tumor necrosis factor-alpha frequently reported in depressed individuals [25]. Our findings suggest that CD3G could serve as a bridge linking immune system dysregulation to the development and persistence of depressive symptoms. Previous studies have not extensively explored CD3G in the context of depression, making this a pioneering discovery.

In the realm of oncology, our pan-cancer analysis reveals that CD3G is upregulated in numerous cancers and is correlated with immune cell infiltration and oncogenic pathways. This finding is consistent with literature suggesting that CD3G, as a component of the T-cell receptor complex, plays a role in T-cell activation and cancer immune surveillance [30]. The upregulation of CD3G in various cancers may indicate its involvement in tumor immune evasion mechanisms. Moreover, the correlation between CD3G expression and immune infiltration across cancers supports the hypothesis that CD3G could serve as a prognostic marker and a potential target for immunotherapy [14].

The dual focus on CD3G in both depression and cancer is a novel aspect of our study. Previous research has typically examined the role of immune genes in either psychiatric disorders or oncology separately. By integrating these fields, our study underscores the importance of immune modulation in both depression and cancer, suggesting potential therapeutic targets that could be leveraged across these conditions. This integrative approach highlights the interconnected nature of immune-related diseases and underscores the importance of holistic biomedical research.

Despite these promising findings, our study has several limitations that should be acknowledged. First, the use of publicly available datasets, while providing a robust sample size, may introduce variability due to differences in data collection methods and patient populations. Second, the cross-sectional nature of the data limits our ability to infer causal relationships between CD3G expression and disease states. Longitudinal studies would be necessary to establish causality and understand the temporal dynamics of immune dysregulation in depression and cancer. Third, while our study identifies CD3G as a potential diagnostic marker, further validation using independent cohorts and prospective clinical trials is essential to confirm its clinical utility. Additionally, the mechanistic insights proposed are based on bioinformatic analyses and require experimental validation to elucidate the precise biological pathways involved.

## Conclusion

In conclusion, our study provides compelling evidence for the role of CD3G as a diagnostic marker for depression and its oncogenic implications. The significant dysregulation in immune cell infiltration and pathway activities highlighted in our findings underscores the critical importance of CD3G in the pathophysiology of both depression and cancer. These insights not only deepen our understanding of the biological underpinnings of these conditions but also open promising new avenues for therapeutic intervention. Targeting CD3G could potentially revolutionize the approach to diagnosing and treating depression and various cancers, offering hope for more effective and personalized medical strategies.

## Data Availability

The data utilized in this study were sourced from the GEO database, accessible at https://www.ncbi.nlm.nih.gov/geo/.
